# Probiotic, Paraprobiotic, and Postbiotic Activities of *Lactiplantibacillus plantarum* KUNN19-2 Against Non-Typhoidal *Salmonella* Serovars

**DOI:** 10.3390/ijms26051821

**Published:** 2025-02-20

**Authors:** Songbo Li, Arishabhas Tantibhadrasapa, Songphon Buddhasiri, Pattarapon Boonpan, Chutikarn Sukjoi, Panupon Mongkolkarvin, Massalin Nakphaichit, Sunee Nitisinprasert, Parameth Thiennimitr

**Affiliations:** 1 Department of Microbiology, Faculty of Medicine, Chiang Mai University, Chiang Mai 50200, Thailand; songbo_l@cmu.ac.th (S.L.); arishabhas.t@gmail.com (A.T.); patponjob@gmail.com (P.B.); sukjoi.chutikarn@gmail.com (C.S.); panuponmkkv@gmail.com (P.M.); 2 Key Laboratory of Basic Research and Transformation of Tumor Immunity and Infectious Diseases, Youjiang Medical University for Nationalities, Baise 533000, China; 3 Research Center for Veterinary Biosciences and Veterinary Public Health, Chiang Mai University, Chiang Mai 50100, Thailand; songphon.bu@cmu.ac.th; 4 Faculty of Veterinary Medicine, Chiang Mai University, Chiang Mai 50100, Thailand; 5 Department of Biotechnology, Faculty of Agro-Industry, Kasetsart University, Bangkok 10900, Thailand; fagimln@ku.ac.th (M.N.); fagisnn@ku.ac.th (S.N.); 6 Specialized Research Unit: Probiotics and Prebiotics for Health, Faculty of Agro-Industry, Kasetsart University, Bangkok 10900, Thailand; 7 Center of Excellence in Microbial Diversity and Sustainable Utilization, Chiang Mai University, Chiang Mai 50100, Thailand; 8 Center of Multidisciplinary Technology for Advanced Medicine, Faculty of Medicine, Chiang Mai University, Chiang Mai 50200, Thailand

**Keywords:** non-typhoidal *Salmonella* (NTS), *Lactiplantibacillus plantarum* KUNN19-2, probiotics, paraprobiotics, postbiotics, heat-killed probiotics, immunomodulation

## Abstract

Non-typhoidal salmonellosis (NTS) caused by multidrug-resistant (MDR) *Salmonella enterica* is a significant public health concern worldwide. Probiotics offer a potential alternative to antibiotics in many infectious diseases, including NTS. However, using living bacteria raises safety concerns in clinical settings, especially in the immunocompromised host. This study compared the anti-*Salmonella* and immunomodulatory effects between viable (probiotics) and heat-killed (paraprobiotics) lactic acid bacteria *Lactiplantibacillus plantarum* KUNN19-2 (KUNN19-2), isolated from Thai-style fermented pork (Nham), against several strains of MDR *Salmonella*. Only viable KUNN19-2 and its cell-free supernatant directly inhibited *Salmonella* growth by spot-on lawn and agar well diffusion assays. A significant reduction in *Salmonella* numbers in the co-culture assay with viable KUNN19-2 was observed at 12–14 h after the incubation. Viable and heat-killed KUNN19-2 exhibited moderate adhesion to human colonic epithelium (T84) cells. Pretreatment with either form of KUNN19-2 enhanced macrophage (RAW264.7) phagocytic activity against *Salmonella* and upregulated pro-inflammatory genes (*Mip-2* and *Nos2*) and anti-inflammatory gene (*IL10*) expression, with viable KUNN19-2 showing a more potent effect. Collectively, viable KUNN19-2 can directly inhibit *Salmonella* growth. However, viable and heat-killed KUNN19-2 can modulate gut immunity against *Salmonella* infection, suggesting that paraprobiotic KUNN19-2 may serve as an alternative treatment against MDR *Salmonella* through host immune modulation.

## 1. Introduction

Non-typhoidal salmonellosis (NTS), caused by oral ingestion of the Gram-negative bacterium *Salmonella enterica* subspecies *enterica*, is among the most common bacterial gastrointestinal tract infections and the third leading cause of diarrheal disease death in humans [1,2]. NTS can range from mild to severe symptoms depending on several factors. Most infected individuals develop a self-limited acute gastroenteritis (diarrhea with high-grade fever). However, the life-threatening form of NTS, termed “invasive NTS”, could occur in some groups of the population, especially immunocompromised hosts and people of extreme ages [3]. Overusing or misusing antibiotics in medicine and agriculture raises the emergence of multidrug-resistant (MDR) *Salmonella* strains worldwide [4,5,6]. There is an alarming concern about the preparedness for alternatives to antibiotics against MDR *Salmonella*.

According to the Food and Agriculture Organization of the United Nations (FAO) and the World Health Organization (WHO), probiotics are defined as “live microorganisms, when administered in adequate amounts, confer a health benefit to the host”. Gram-positive bacteria in the genus *Lactobacillus* are widely used in medicine, the food industry, and agriculture. The beneficial effects of probiotic *Lactiplantibacillus plantarum* (formerly named *Lactobacillus plantarum*) against several bacterial infections have already been extensively investigated. Oral administration of *Lactiplantibacillus* probiotics prevents pathogenic bacteria colonization at the host mucosal surfaces or colonization resistance. Several mechanisms of probiotics *Lactiplantibacillus* in inhibiting the growth of gut pathogenic bacteria in the host gastrointestinal tract have already been reported. The colonization of viable probiotics or balanced gut microbial communities in the gastrointestinal tract prevents the adhesion and invasion of enteropathogens by niche modification or pre-emption [7]. Some strains of *Lactiplantibacillus* spp. secrete antimicrobial substances and metabolites, especially the fermentation end product lactic acid, that directly inhibit the growth of enteropathogens. Moreover, probiotics can also indirectly inhibit the growth of harmful bacteria by manipulating host gut innate and adaptive immunity [8]. For instance, *L. plantarum* 1201 alleviates gut inflammation in mice infected with *Salmonella enterica* serovar Typhimurium by suppressing the *Salmonella*-activated inflammatory pathways [9].

Even though the large body of evidence supports the beneficial effects of using live probiotics in humans, concerns about viable probiotics in clinical use have also been raised [10]. Large consumption of live microorganisms might increase the risk of sepsis or uncontrollable inflammatory response in immunocompromised individuals, young children, or the elderly. The expression of hidden virulence factors in probiotic strains might occur when given to the susceptible hosts. Moreover, there is an increased chance of antibiotic resistance-gene transfer between the viable probiotics and the resident gut microbiota, leading to the spread of MDR genes among host gut microbiota.

Hence, using a non-viable form or dead compartments of probiotics, termed “paraprobiotics”, has become an interesting topic in probiotics research [10,11,12,13]. Some studies showed a highly beneficial effect of using paraprobiotics over probiotics. Heat-killed *L. rhamnosus* GG (LGG) exhibits a higher anti-inflammatory effect on gut epithelium than its viable form [14]. On the other hand, heat-killed *Lactobacillus* spp. inhibit the growth of several enteropathogens lower than that of its viable form [15]. Few mechanisms, such as the preformed metabolites from viable probiotics or host immunomodulation from the recognition between pattern recognition receptors on host immune cells and microbial-associated molecular patterns on bacterial cells, were proposed as antagonistic effects of the paraprobiotics. However, the strain-specific effect of probiotics or paraprobiotics plays a major role in the outcome [16].

The probiotic *L. plantarum* strain KUNN19-2 (KUNN19-2) (formerly classified as *L. johnsonii* KUNN19-2) was originally isolated from Thai-style fermented pork (Nham) and exhibited an immunomodulatory effect in mice [17]. Heat-killed KUNN19-2 increased immunoglobulin G production in mouse splenocytes by an ex vivo test. Nonetheless, a comparison between viable and heat-killed KUNN19-2 and an investigation of their anti-*Salmonella* and immunomodulatory properties have never been performed. Here, we tested the direct *Salmonella* growth inhibition and indirect immunomodulatory effects of probiotic (viable or live) and paraprobiotic (heat-killed or dead) KUNN19-2 against clinically isolated MDR *Salmonella* using cell culture models.

## 2. Results

### 2.1. Clinical Characteristics, MDR Phenotype, and Virulence Genes of Clinically Isolated Non-Typhoidal Salmonella Serovars

All six *Salmonella enterica* strains (STMC58, 081, 101, 103, 166, and 177) were isolated from a stool bacterial culture of acute gastroenteritis patients admitted to Maharaj Nakorn Chiang Mai Hospital (MNCMH) during the year 2017–2019 (Supplementary Tables S1 and S2). Clinical parameters, including age, gender, sepsis status, hematocrit (Hct), and white blood cell count, were reported. Five *Salmonella* isolates (STMC58, 81, 101, 103, and 166) have MDR phenotype by the disc assay (Supplementary Table S3). However, STMC177 (serovar Enteritidis) is resistant only to nalidixic acid. The presence of four key virulence genes (*spvB*, *sseI*, *sodCI*, and *rpoS*) in clinical *Salmonella* isolates was detected using PCR, with primers detailed in Supplementary Table S4. The distribution of these four virulence genes among the clinical strains is shown in Supplementary Table S2. The results revealed that all five clinical isolates contained the *rpoS* gene. The *spvB* was only detected in STMC177, harboring the *spvB*-*sseI*-*sodCI*-*rpoS* gene pattern. The *sseI*-*sodCI*-*rpoS* pattern was identified only in STMC58. STMC81 and STMC166 exhibited the *sodCI*-*rpoS* pattern. STMC101 and STMC166 harbored only the *ropS*.

### 2.2. No Morphological Change in Paraprobiotic KUNN19-2

The morphology of paraprobiotic (heat-killed) versus probiotic (viable) KUNN19-2 was observed by light (Supplementary Figure S1A,B) and scanning electron microscope (Supplementary Figure S1C,D). Our data show that inactivating KUNN19-2 by an autoclaving process (121 °C for 15 min at 15 psi) kills the bacterium without altering its morphology observed under a scanning electron microscope. The short-rod shape with rounded ends of viable and heat-killed KUNN19-2 was still observed with an insignificant difference.

### 2.3. Direct Anti-Salmonella Effect of Viable Probiotic KUNN19-2

**Figure 1:**
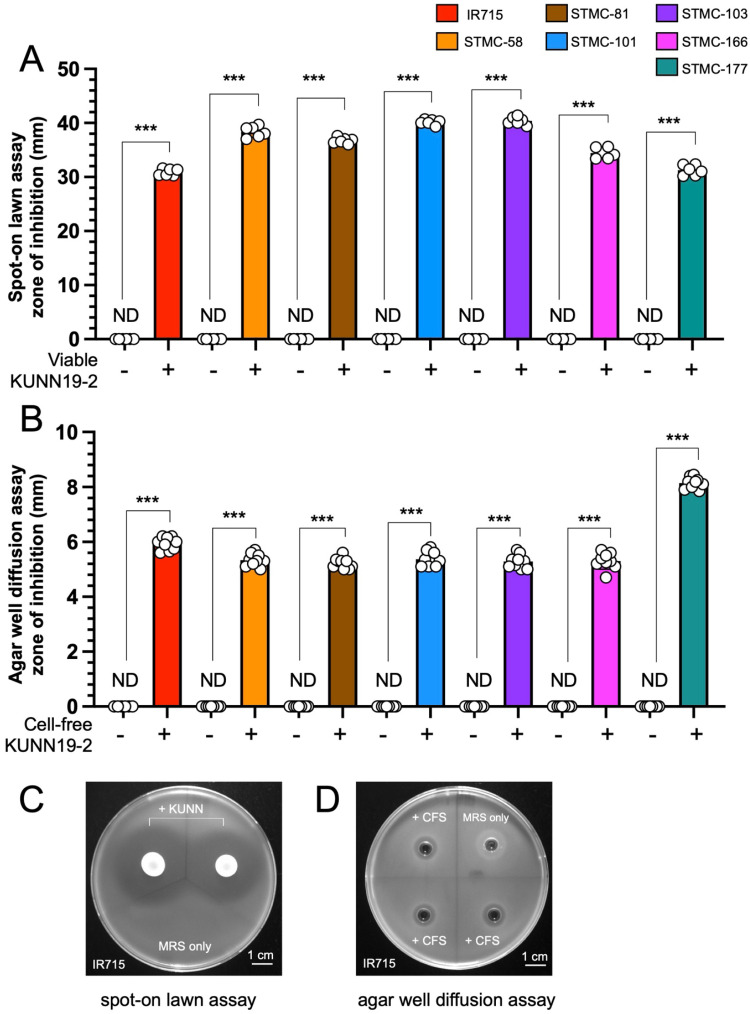
*L. plantarum* KUNN19-2 directly inhibits the growth of several strains of MDR *Salmonella*. Zone of inhibition (mm) from spot-on lawn assay (**A**) and agar well diffusion assay (**B**). Bars represent the geometric mean, with geometric standard deviation of at least three biological replicates. *** *p* < 0.001; ND, not detectable (clear zone diameter < 1 mm). MRS broth was used as a negative control. The representative picture of the spot-on lawn assay (**C**) and agar well diffusion assay (**D**).

The spot-on lawn and agar well diffusion assays were used to determine the direct anti-*Salmonella* effect of both viable (whole cell) and cell-free supernatant (CFS) or postbiotic of KUNN19-2, respectively (Figure 1). Viable and CFS forms of KUNN19-2 significantly inhibited the growth of all *Salmonella* strains used in this study. The diameter (mm) of the inhibition zone around the spot or well was measured (Figure 1A,B, respectively). Representative pictures of the spot-on lawn and agar well diffusion assays are shown in Figure 1C,D, respectively. Interestingly, only CFS from viable KUNN19-2 generated the inhibition zone around the well in an agar well diffusion assay (Supplementary Figure S2).

### 2.4. Viable Probiotic KUNN19-2 Inhibits Salmonella Growth in Co-Culture Media

**Figure 2:**
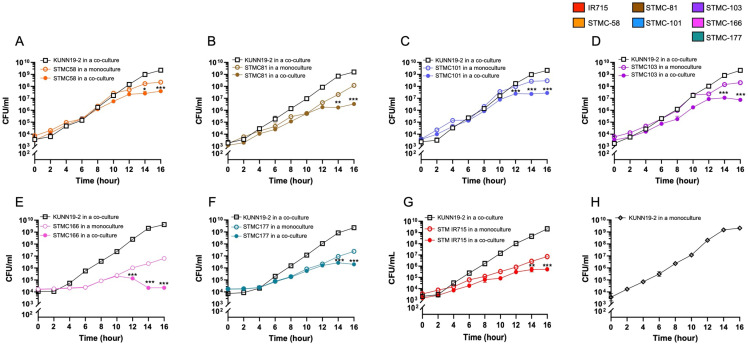
Anti-*Salmonella* effect of probiotics *L. plantarum* KUNN19-2 in co-culture media. The co-culture medium (1:1 volume of double strength MRS and MH broth) and single medium (MRS or MH broth) were used to observe the kinetics of the anti-*Salmonella* effect of viable KUNN19-2. The growth of clinically isolated *Salmonella* strains (STMC58, 81, 101, 103, 166, and 177) and STM IR715 was significantly inhibited by viable KUNN19-2 (**A**–**G**). The single growth of KUNN19-2 was also shown (**H**). Bars represent the geometric mean, with geometric standard deviation of at least three independent experiments. * *p* < 0.05; ** *p* < 0.01; *** *p* < 0.001.

Next, we investigated the competitive growth between viable probiotic KUNN19-2 and all *Salmonella* isolates. Equal (1:1) mixtures of KUNN19-2 and *Salmonella* isolates (IR715, STMC58, 81, 101, 103, 166, and 177) were statically grown in a double-strength (2X) co-culture (De Man–Rogosa–Sharpe (MRS) and Mueller Hinton (MH)) broth and incubated at 37 °C without shaking. The cfu/mL of each bacterium was calculated at the indicated time points (Figure 2). At 10–12 h post-inoculation, a significant reduction in *Salmonella* numbers in co-culture medium was observed, but not in a mono-culture medium. Viable KUNN19-2 demonstrated a similar pattern of anti-*Salmonella* effect with all tested *Salmonella* isolates (Figure 2A–G). Growth of KUNN19-2 alone in a co-culture medium was also shown (Figure 2H).

### 2.5. Viable and Heat-Killed KUNN19-2 Adhere to Human Colonic Epithelium

**Figure 3:**
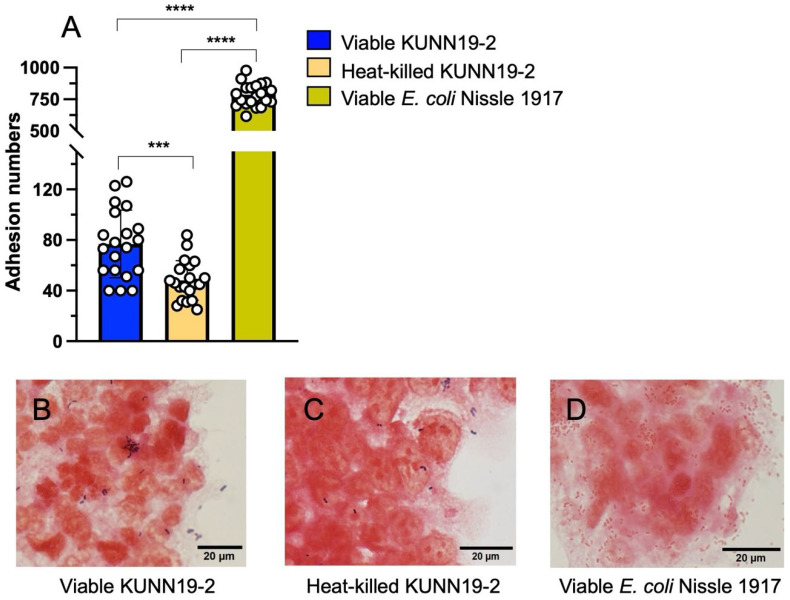
Adhesiveness of viable and heat-killed KUNN19-2 to human colonic epithelium. Adhesion numbers were counted by the presence of adherent *Lactobacilli* in twenty random fields of an oil-immersion objective lens (100×). Viable *L. plantarum* KUNN19 (**A**, blue bar) significantly adheres to human colonic epithelium cells (T84 cells) higher than its heat-killed form (**A**, orange bar). However, both are significantly lower in adhesive properties on T84 cells compared to that of *E. coli* Nissle (EcN) 1917 (**A**, yellow bar). Bars represent the geometric mean, with geometric standard deviation of at least three independent experiments. *** *p* < 0.001; **** *p* < 0.0001. Representative pictures of a methanol-fixed Gram staining of viable KUNN19-2, heat-killed KUNN19-2, and EcN 1917 on T84 cells are shown in (**B**–**D**), respectively. The indicated scale bar is 20 µm.

The ability of probiotic bacteria to adhere to the host gut epithelium is among the most significant properties for use in clinical settings. We investigated the adhesion efficacy of both viable and heat-killed KUNN19-2 onto human colonic epithelium (T84) cells compared to the prototypic gut probiotics *Escherichia coli* Nissle (EcN) 1917. By using a methanol-fixed Gram’s staining method, we found that viable KUNN19-2 was more adhesive to T84 cells than its heat-killed form but still lower than EcN 1917 (Figure 3A–D). Nonetheless, both viable and heat-killed KUNN19-2 significantly showed a moderate gut adhesion property. The adhesion numbers are approximately 76.8 and 48.0 for viable and heat-killed KUNN19-2, respectively.

### 2.6. Dose-Dependent Cytotoxicity of Viable KUNN19-2 on Human Colonic Epithelium and Murine Macrophage

**Figure 4:**
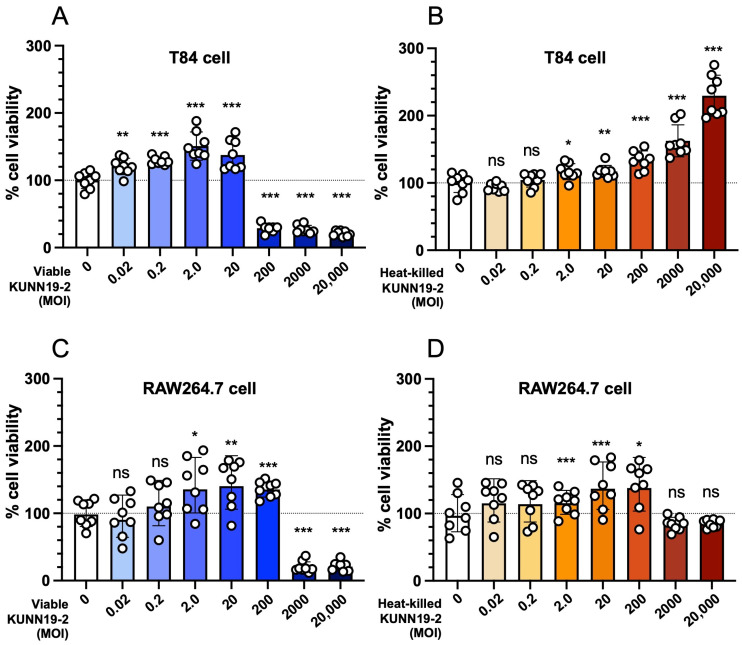
Dose-dependent cytotoxicity of viable and heat-killed *L. plantarum* KUNN19-2 on human colonic epithelium and mouse macrophage by an MTT assay. Different doses of viable or heat-killed KUNN19-2, ranging from the multiplicity of infection (MOI) 0 to 20,000, were used to investigate the optimal dose for the cell culture assay. Human colonic epithelial cells (T84) were treated with viable (**A**) or heat-killed (**B**). Mouse macrophages (RAW264.7) were treated with viable (**C**) or heat-killed (**D**) KUNN19-2. Bars represent the geometric mean, with geometric standard deviation of three independent experiments. * *p* < 0.05; ** *p* < 0.01; *** *p* < 0.001; ns, a non-statistically significant difference.

The MTT assay was performed to find the non-toxicity doses of viable and heat-inactivated KUNN19-2 for subsequent cell culture experiments. Human colonic epithelium (T84) and mouse macrophage (RAW264.7) cells were pretreated with different multiplicities of infections (MOIs), ranging from 0 to 20,000, at 37 °C, with 5% CO_2_, for 24 h. Our data show the dose-dependent effect of only viable but not heat-killed KUNN19-2. In the viable KUNN19-2-treated groups, T84 and RAW264.7 cell viability decreased at MOI 200 and 2000, respectively (Figure 4A,C). Interestingly, the heat-killed KUNN19-2 does not reduce the viability of both cells compared to the untreated group (Figure 4B,D). MOI 20, the highest MOI that does not reduce cell viability, was selected for further use.

### 2.7. Pretreatment with Both Viable and Heat-Killed KUNN19-2 Enhances Macrophage-Killing Activity and Proinflammatory Gene Expressions After Salmonella Infections

**Figure 5:**
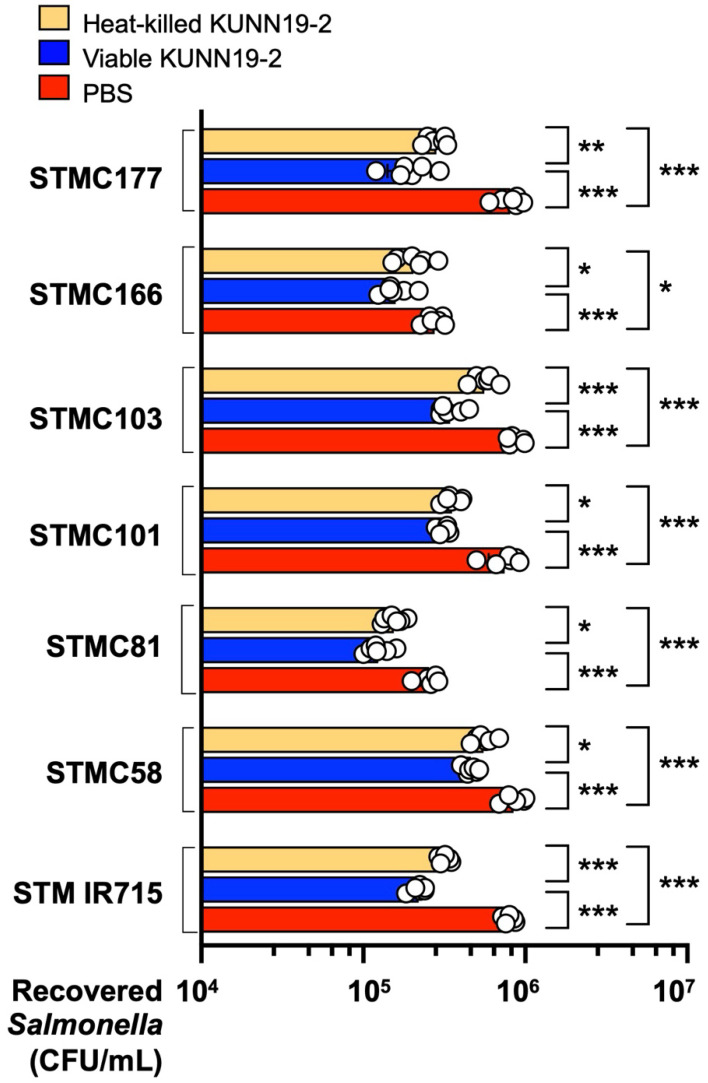
Viable and heat-killed KUNN19-2 enhance macrophage-killing activity against *Salmonella*. Mouse macrophage (RAW264.7) cells were pretreated with heat-killed (orange bar) or viable (blue bar) KUNN19-2 or PBS as a control (red bar) for 24 h before *Salmonella* infection. At 1 h post-infection with different *Salmonella* isolates (STMC177, 166, 103, 101, 81, 58, and IR715), macrophages were lysed, and the recovered *Salmonella* numbers (CFU/mL) were enumerated. Bars represent the geometric mean, with a geometric standard deviation of at least three independent experiments. * *p* < 0.05, ** *p* < 0.01, *** *p* < 0.001.

To compare the immunomodulatory effect of both viable and heat-killed KUNN19-2 on macrophages infected with different strains of *Salmonella*, the gentamicin protection assay was performed. Our data show that pretreatment with viable or heat-killed KUNN19-2 for 24 h before *Salmonella* infection significantly enhances the phagocytic activity of RAW264.7 compared to the untreated group (Figure 5). Reduced numbers of intracellular *Salmonella* were shown in the KUNN19-2-treated groups. Interestingly, the viable KUNN19-2 (Figure 5, blue bar) induced more of an anti-*Salmonella* effect than the heat-killed KUNN19-2 (Figure 5, orange bar).

**Figure 6:**
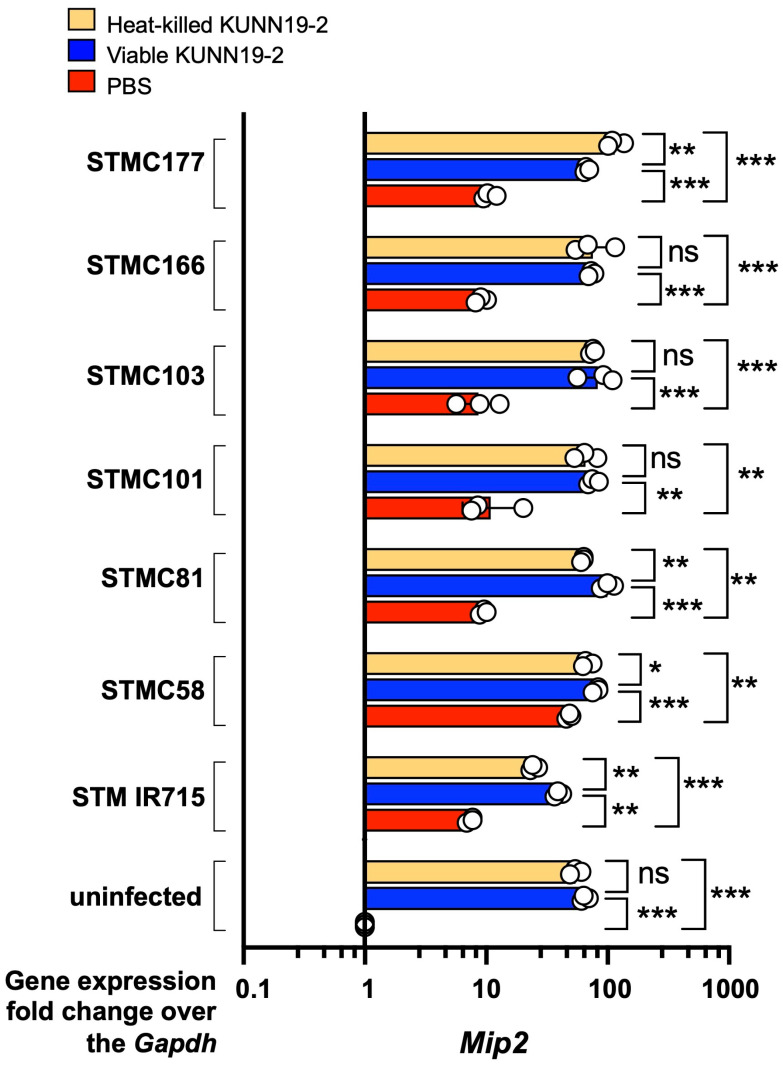
Viable and heat-killed KUNN19-2 increase *Mip-2* gene expression in macrophages. Pretreatment with heat-killed (orange bar), viable (blue bar) KUNN19-2 or PBS (red bar) on mouse macrophage RAW264.7 cells for 24 h enhanced the *Mip-2* expression at 1 h post-infection regardless of *Salmonella* infection. PBS, phosphate-buffered saline. Bars represent the geometric mean with geometric standard deviation of at least three independent experiments. * *p* <0.05, ** *p* < 0.01, *** *p* < 0.001; ns, a non-statistically significant difference.

**Figure 7:**
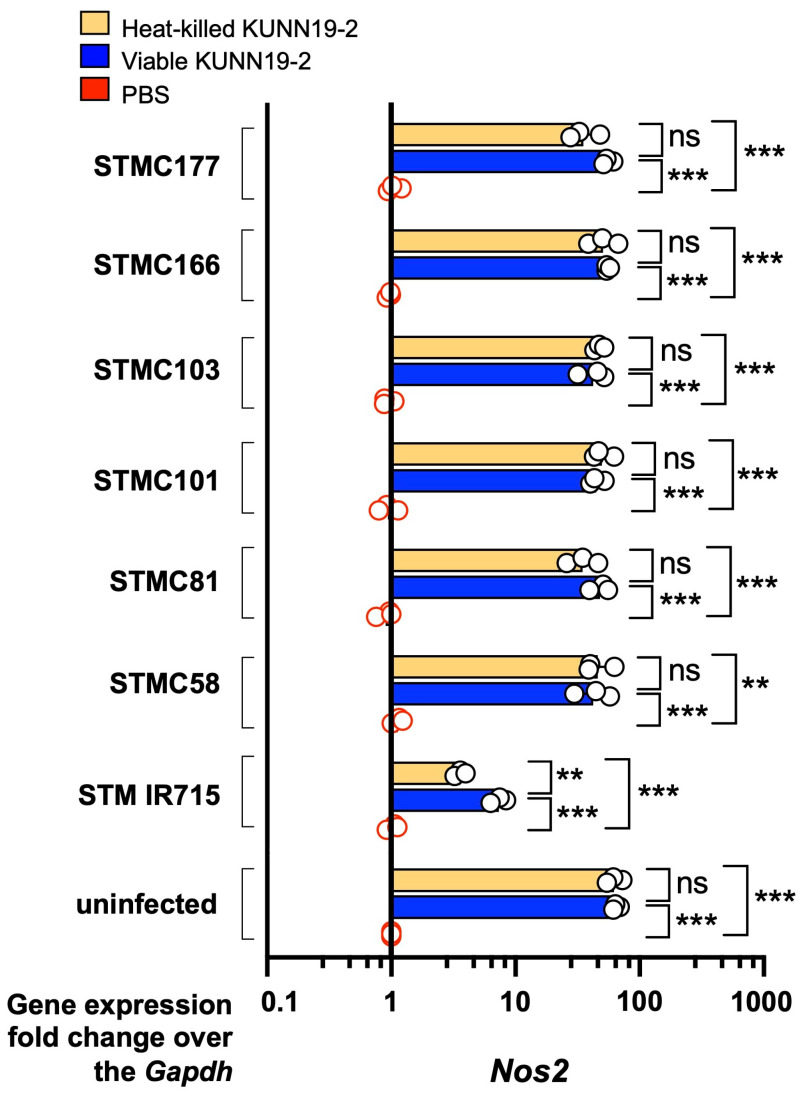
Viable and heat-killed KUNN19-2 increase *Nos2* gene expression in macrophages. Pretreatment with heat-killed (orange bar), viable (blue bar) KUNN19-2 or PBS (red circle) on mouse macrophage RAW264.7 cells for 24 h enhanced the expression of *Nos2* at 1 h with or without *Salmonella* infection. No significant difference in *Nos2* expression between viable and heat-killed KUNN19-2. PBS, phosphate-buffered saline. Bars represent the geometric mean, with geometric standard deviation of at least three independent experiments. ** *p* < 0.01; *** *p* < 0.001; ns, a non-statistically significant difference.

Next, we compared pro-inflammatory gene (*Mip2* and *Nos2*) expressions at 1 h post-*Salmonella* infection in macrophages with or without KUNN19-2 pretreatment. Our data show that pretreatment with either viable or heat-killed KUNN19-2 significantly increases the expression of *Mip2*, encoding for macrophage inflammatory protein (Mip)-2 (Figure 6). There was no significant difference in *Mip-2* expression between viable and heat-killed KUNN19-2 in the uninfected macrophages. However, there was a decreased *Mip-2* expression in macrophages infected with STMC81, STMC58, and STM IR715. The heat-killed KUNN19-2 upregulated *Mip-2* expression in macrophages infected with STMC177. Both viable and heat-killed KUNN19-2-pretreated macrophages significantly increased the expression of *Nos2*, encoding for inducible nitric oxide synthase (iNOS) (Figure 7). There was no significant difference in *Nos2* expression between viable and heat-killed KUNN19-2-pretreated uninfected macrophages.

**Figure 8:**
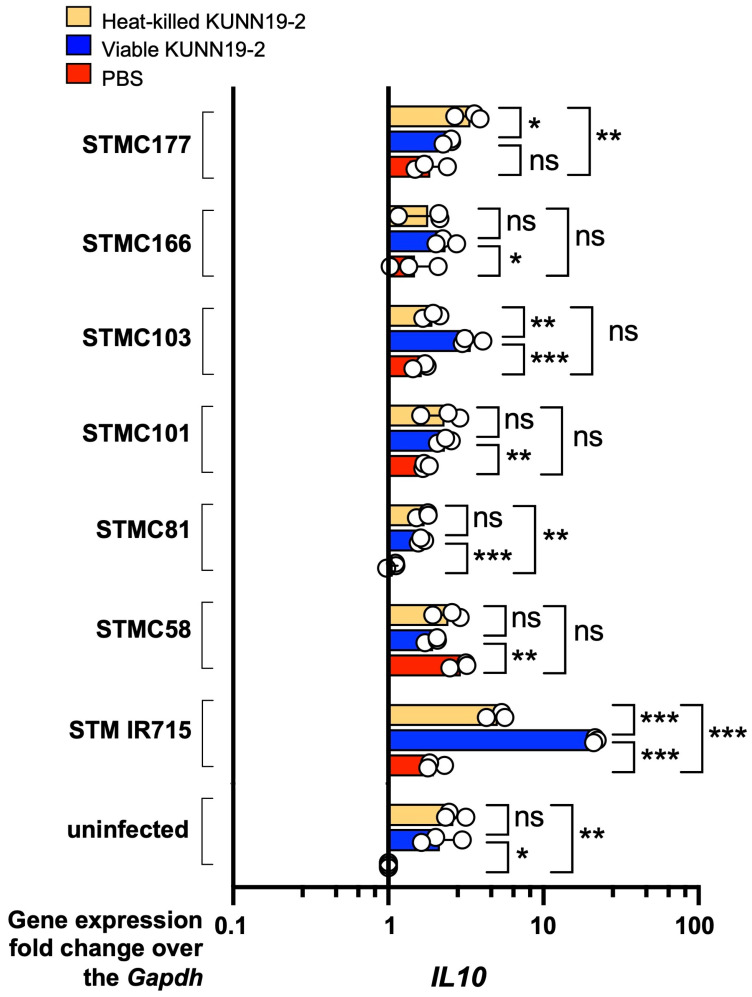
Viable and heat-killed KUNN19-2 increase *IL10* gene expression in macrophages. Pretreatment with heat-killed (orange bar), viable (blue bar) KUNN1-2 or PBS (red bar) on mouse macrophage RAW264.7 cells for 24 h upregulated *IL10* expression at 1 h with or without *Salmonella* infection. A variation in *IL10* activation of viable and heat-killed KUNN19-2 was found. PBS, phosphate-buffered saline. Bars represent the geometric mean, with a geometric standard deviation of at least three independent experiments. * *p* <0.05; ** *p* < 0.01; *** *p* < 0.001; ns, a non-statistically significant difference.

We also detected the expression of the *IL10* gene, encoding for anti-inflammatory cytokine interleukin (IL)-10. Our data show a variation in the *IL10* induction effect of viable and heat-killed KUNN19-2 among different strains of *Salmonella* infection (Figure 8). Viable KUNN19-2 upregulated *IL10* expression in macrophages infected with IR715. However, there was no significant difference in *IL10* expression between viable and heat-killed KUNN19-2 pretreated macrophages infected with STMC166, 101, 81, and 58. On the other hand, there was a decreased *IL10* expression in heat-killed KUNN19-2-pretreated macrophages infected with STMC103 and IR715 compared to the viable form-treated group.

## 3. Discussion

Despite evidence supporting the beneficial roles of viable probiotic *Lactobacillus* in human bacterial infections, including NTS, the infrequent but serious adverse effect has been occasionally reported [18,19,20]. Concerns about using live microorganisms in immunocompromised patients have been raised. Three Gram-positive bacteria probiotics (*Lacticaseibacillus rhamnosus* GG, *Lactiplantibacillus plantarum*, and *Lacticaseibacillus paracasei*) had been reported as possible causative agents of bacteremia in patients who were orally taking live *Lactobacilli* [21]. Patients with severe conditions, immune suppression, or central venous catheterization, and pre-term babies, are the high-risk groups for this rare but life-threatening condition.

Recently, the advantages of the inactivated components of probiotics (paraprobiotics) and their metabolites (postbiotics) as functional foods have been reviewed [12]. Here, we compared the anti-*Salmonella* and immunomodulatory effects between probiotic (viable) KUNN19-2 and its paraprobiotic (heat-killed) forms against clinically isolated MDR *Salmonella*. *Salmonella* strains were collected from the stool cultures of acute gastroenteritis patients admitted to MNCMH during 2017–2019, and their clinical characteristics were shown. Four out of six patients are in the extreme ages and have elevated levels of total leukocyte counts. Five serovars, namely 1,4,[5],12:i-, Typhimurium, Krefeld, Rissen, and Enteritidis of *S. enterica*, were identified by WGS. A random pattern of four *Salmonella* virulence genes (*spvB*, *sseI*, *sodCI*, and *rpoS*) was found. All isolates contain *the rpoS* gene, encoding for the RNA polymerase sigma factor RpoS, the master regulator of the general stress response in *Salmonella*. *RpoS* also controls the expression of numerous genes involved in survival under adverse conditions, such as oxidative stress, acidification, and starvation [22]. Other virulence genes (*spvB*, *sseI*, *sodCI*) were randomly distributed among these six isolates. The MDR phenotype of *Salmonella* was found in all *Salmonella* isolates except STMC177 (serovar Enteritidis) isolated from a 19-year-old man. These results are similar to those of the previous report [23].

The appropriate heat-inactivation process in making paraprobiotics is essential to maintaining their integral morphology and their biological activities when interacting with the host environment [24]. Our data show no significant change in the morphology of heat-killed KUNN19-2 compared to its viable form under light and scanning electron microscopes. Viable KUNN19-2 can significantly inhibit the growth of all tested *Salmonella* isolates. However, only CFS from viable KUNN19-2 can inhibit *Salmonella* growth. Our findings are in accordance with the previous study illustrating that only viable KUNN19-2 and its CFS, but not the heat-killed form of *Lactobacillus* probiotics, exhibit a direct antimicrobial effect against several human oral pathogens [25]. The inhibition zone in agar well diffusion assay in our study ranged from 5.00 to 8.40 mm, thus differing from that in the previous report (10.0 to 17.2 mm) [26]. There was a significant reduction in *Salmonella* numbers in the co-culture compared to the monoculture assay, which started 12–14 h after the incubation. This reduction in *Salmonella* is probably due to the strong acidity of the culture medium. However, these time points differed from the previous report showing that live probiotic *Ligilactobacillus salivarius* inhibits the growth of *S.* Enteritidis, *S.* Infantis, and *S.* Kentucky ST198 at 4–8 h after inoculation in the co-culture assay [27]. Moreover, we observed the inhibitory effect of viable probiotic KUNN19-2 when it reached a density of about 10^6^–10^7^ cfu/mL. These indicate the strain-specificity of probiotics and the susceptibility of pathogens to the direct growth inhibitory effect.

Adhesion to intestinal epithelium is one of the significant properties of probiotics in preventing enteropathogen colonization. Here, we found that viable and heat-killed KUNN19-2 can adhere to human colonic epithelium. However, the adhesion was decreased in the heat-killed compared to the viable KUNN19-2. The adhesion numbers of KUNN19-2 on human colonic epithelium were also lower than those of probiotics EcN 1917. These could result from the heat-sensitive proteinaceous molecules on bacterial cells being destroyed during the heat-inactivation process [28]. On the contrary, the heat-killed *L. rhamnosus* 3698 and *L. farciminis* 3699 demonstrated a higher adhesion property than their viable forms on Caco-2 cells [29]. These inconsistent outcomes indicate the strain-specific effect of the probiotics on gut adhesion between their live and dead form.

High doses (MOI >= 200) of viable KUNN19-2 significantly reduced the cell viability of T84 and RAW264.7 cells. Interestingly, the reduction in cell viability was not found in the heat-killed KUNN19-2. This is probably due to a very high concentration of metabolites produced from the viable KUNN19-2. Pretreatment macrophages with viable or heat-killed KUNN19-2 significantly reduced the *Salmonella* numbers compared to the untreated group. This indicates the increased phagocytic activity of macrophages after the activation with either live or dead KUNN19-2. However, the enhanced phagocytic activity was higher in the viable KUNN19-2 treated than the heat-killed KUNN19-2 group.

Viable and heat-killed KUNN19-2 enhanced the expressions of proinflammatory-related genes *Mip-2* and *Nos2* in RAW264.7 cells regardless of *Salmonella* infection. MIP-2, the important chemoattractant for phagocytic cell infiltration at the infected site [30]. Our study shows a variation in macrophage *Mip-2* activation caused by the heat-killed KUNN19-2 among different *Salmonella* isolates. Pretreatment with heat-killed KUNN19-2 enhanced *Mip-2* expression only in STMC177 infection but not with other *Salmonella* infections. There was no significant difference in macrophage *Mip-2* upregulation by viable and heat-killed KUNN19-2 in STMC166, STMC103, and STMC101 infections. Nonetheless, the heat-killed KUNN19-2 induced less *Mip-2* expression than its viable form in STMC81-, STMC58-, and IR715-infected macrophages. There was no *Nos2* gene upregulation in *Salmonella*-infected RAW264.7 at 1 h post-infection, but pretreatment with either viable or heat-killed KUNN19-2 significantly enhanced *Nos2* expression in macrophages. These observations suggest the potential of using heat-killed KUNN19-2 for macrophage activation. Heat-killed KUNN19-2 upregulated *Nos2* expression indifferently from its viable form in all clinical *Salmonella* infections.

Some probiotics or paraprobiotics can induce anti-inflammatory cytokine IL-10 to maintain host gut homeostasis [31]. We found that pretreatment with probiotic or paraprobiotic KUNN19-2 significantly increased *IL10* expression in *Salmonella*-infected macrophages. However, the magnitude of the *IL10* activation varied among the different isolates of *Salmonella*. The heat-killed KUNN19-2-activated *IL-10* expression in macrophages was not different from its viable form. Inconsistent with our findings, probiotics *L. plantarum* BFE 1685 and *L. johnsonii* BFE 6128 did not modulate the expression of IL-1, IL-6, IL-10, and monocyte chemoattractant protein-1 (MCP-1) from HT29 intestinal epithelial cells [32]. However, the secreted cytokines in the gut epithelium (T84) or macrophage cell culture supernatant, after being challenged with KUNN19-2, were not detected in this study.

## 4. Materials and Methods

### 4.1. Ethical Approvals

The retrospective study of medical records of six acute gastroenteritis patients admitted to MNCMH was performed with the approval of the Research Ethics Committee (Approval No. 135/2024) and Institutional Biosafety Committee, Faculty of Medicine, Chiang Mai University (Approval No. 02024/2566).

### 4.2. Bacterial Strains, Cultivation, and Heat-Inactivated Condition

All bacterial strains used in this study are listed in Supplementary Table S1. Probiotic *L. plantarum* KUNN19-2, originally isolated from Thai-style fermented pork (Nham), was statically cultured in 5 mL of de Man, Rogosa, and Sharpe (MRS) broth (10 g/L proteose peptone No. 3, 10 g/L beef extract, 5 g/L yeast extract, 20 g/L dextrose, 1 g/L polysorbate 80, 2 g/L ammonium citrate, 5 g/L sodium acetate, 0.1 g/L magnesium sulfate, 0.05 g/L manganese sulfate, and 2 g/L dipotassium phosphate) (Difco, MD, USA) at 37 °C in a microaerophilic condition (without shaking) for 24 h, unless stated otherwise. For heat inactivation, the KUNN19-2 overnight culture was centrifuged at 4000 g for 10 min to collect the pellet and washed twice with sterile phosphate-buffered saline (PBS). Then, bacterial pellet was heat-killed by autoclaving at 121 °C for 15 min under 15 psi in a 50 mL conical tube and kept at −20 °C until use. *Salmonella enterica* strains were cultured in Luria-Bertani (LB) broth (10 g/L tryptone, 5 g/L yeast extract, and 10 g/L NaCl) (Difco, MD, USA) with shaking at 37 °C for 16–18 h. All bacterial cultures were performed at the ambient atmospheric gas conditions.

### 4.3. Antibiotic Susceptibility Test of Clinical Salmonella Isolates

The Kirby–Bauer method was used to determine the antibiotic susceptibility of all six clinical isolates, as previously described [33]. Briefly, *Salmonella* colonies were resuspended in 3 mL PBS until the turbidity of the suspension reached 0.5 McFarland and streaked over the surface of MH agar (approximately 4 mm depth). A total of 15 antibacterial agents in 9 categories were used (Supplementary Table S3). We placed 15 antimicrobial disks (Oxoid, UK) of streptomycin (S; 10 µg), cephazolin (KZ; 30 µg), cefuroxime (CXM; 30 µg), cefotaxime (CTX; 30 µg), ceftriaxone (CRO; 30 µg), ceftazidime (CAZ; 30 µg), cefepime (FEP; 30 µg), nalidixic (NA acid; 30 µg), aztreonam (ATM; 30 µg), amoxicillin/clavulanic acid (AMC; 30 µg), doxycycline (DO; 30 µg), tetracycline (TE; 30 µg), trimethoprim/sulfamethoxazole (SXT; 25 µg), ampicillin (AMP; 10 µg), and ciprofloxacin (CIP; 5 µg) on the agar and measured the clear zone diameter at 18 h after incubation at 37 °C. Antibiotic susceptibility was interpreted following the 2020 Clinical & Laboratory Standards Institute (CLSI) guideline. R >= 3 categories were considered MDR.

### 4.4. Salmonella Serovar Determination by Whole Genome Sequencing

Whole-genome sequencing (WGS) was used to determine the serovar of six *Salmonella enterica* strains. *Salmonella* genomic DNA was extracted using the Qiagen DNeasy UltraClean Microbial Kits (Qiagen, Valencia, CA, USA) according to the manufacturer’s protocol. The bacterial genome sequencing was performed using the Illumina platform. The paired-end 2 × 150-bp sequencing libraries were constructed using the NEBNext Ultra II DNA Library Prep kit and sequenced with an Illumina NovaSeq sequencer (Illumina, Inc., San Diego, CA, USA). Genomes were assembled using SPAdes 3.15.4 [34]. The assemblies were assigned for *Salmonella* serovar using SeqSero v1.2 [35].

### 4.5. Morphological Study of Viable and Heat-Killed KUNN19-2

The morphology of viable and heat-killed KUNN19-2 was Gram-stained and observed under the light microscope (Olympus, BX63 with DP80 camera, Olympus Corporation, Tokyo, Japan) and the scanning electron microscope (Jeol brand, JSM-6610LV, JEOL Ltd., Tokyo, Japan). In brief, viable and heat-killed KUNN19-2 solutions were filtered through a 0.22 μm pore-size polycarbonate membrane. The membrane-attached bacterial cells were washed twice and resuspended in 0.1 M PBS (pH 7.2), and then they were fixed with 2.5% (*w*/*v*) glutaraldehyde in PBS for 2 h. After rinsing twice with PBS, cells were dehydrated in ascending ethanol concentrations (50%, 70%, 85%, 95%, and 100% *v*/*v*) and dried by a critical point dryer (Quorum brand, model K850, A Judges Scientific plc Company, London, UK). Dried cells were coated with gold (Au) by rotary pumped coater (Quorum brand, model Q150rs, A Judges Scientific plc Company, London, UK).

### 4.6. Spot-On the Lawn and Agar Well Diffusion Assays

For the spot-on the lawn assay, 20 µL of viable KUNN19-2 overnight culture (approximately 2 × 10^7^ CFU) was dropped on the MRS agar and incubated at 37 °C for 12 h, as previously described [36]. Briefly, 200 µL of *Salmonella* overnight culture (approximately 2 × 10^7^ CFU) was mixed with melted LB agar (0.75%) and poured over the plate to create the bacterial lawn. Plates were incubated at 37 °C for 16 h to observe an inhibition zone (more than 1 mm around the KUNN19-2 spot was considered as positive). For the agar well diffusion assay, KUNN19-2 overnight culture was centrifuged at 5000× *g* for 10 min at 4 °C. The cell-free supernatant (CFS) of KUNN19-2 was collected and filtered through a 0.45 µm pore size polyethersulfone (PES) membrane (Whatman, Puradisc). Then, 200 µL of *Salmonella* overnight culture was mixed with melted LB agar (0.75%) to create a bacterial lawn with 6 mm diameter wells. Each well was filled with 60 µL CFS and incubated at 37 °C for 16 h to observe the inhibition zone (more than 1 mm around the well was considered positive).

### 4.7. Viable Probiotic KUNN19-2 and Salmonella Co-Culture Assay

An equal mixture (1:1) of viable probiotic KUNN19-2 and *Salmonella* was used for the co-culture assay. Briefly, 100 µL of KUNN19-2 and *Salmonella* (approximately 10^4^ cfu/mL of each) was inoculated into 10 mL of the co-culture broth (1:1 volume of double strength MRS and MH broth) and incubated at 37 °C for 16 h, without shaking. The recovered numbers of KUNN19-2 and *Salmonella* were enumerated on the appropriate agar at the indicated time points by a ten-fold serial dilution technique.

### 4.8. Cell Culture

Human colonic epithelium T84 (ATCC; CCL-248) and murine macrophage RAW264.7 (ATCC; TIB-71) cells were purchased from the American Type Culture Collection (ATCC, Manassas, VA, USA). Each cell was propagated following the manufacturer’s instructions. In brief, T84 cells were cultured in Dulbecco’s Modified Eagle Medium (DMEM)/F-12 medium (Cytiva, Utah, USA) supplemented with 8% heat-inactivated fetal bovine serum (FBS) (Cytiva, Utah, USA), 0.1 mM non-essential amino acids (Cytiva, Utah, USA), 100 U/mL penicillin, and 0.1 g/mL streptomycin (Cytiva, Utah, USA) in a T75 flask. For RAW264.7 cells, Dulbecco’s Modified Eagle Medium (DMEM, Cytiva, Utah, USA) supplemented with 10% heat-inactivated FBS, 100 U/mL penicillin, and 0.1 g/mL streptomycin was used. Both cells were incubated at 37 °C, with 5% CO_2_, and at 95% relative humidity.

### 4.9. Adhesion Assay of Viable and Heat-Killed KUNN19-2

T84 cells were seeded in a 6-well plate (10^6^ cells per well). Cells were washed twice with sterile PBS before adding 3 mL DMEM/F-12 and incubated at 37 °C with 5% CO_2_ for 24 h. Then, cells were pretreated and incubated with viable or heat-killed KUNN19-2 and viable EcN 1917 as a positive control (MOI 100) at 37 °C with 5% CO_2_ for 24 h. Non-adherent bacteria were washed off with sterile PBS twice. Then, all cells were fixed with absolute methanol (AR grade, RCI Labscan, Ireland) and incubated for 10 min at room temperature. After the removal of methanol by pipetting, cells were stained with Gram’s stain kit (BD Bioscience, Franklin Lakes, NJ, USA) and observed under the light microscope (Olympus BX63) equipped with an Olympus DP80 CCD camera (Olympus, Tokyo, Japan). The adherent bacterial cells in twenty random high-power fields were counted and reported as adhesion numbers. Adhesion numbers less than 40, between 41 and 100, and more than 100 were assigned as non-adhesive, adhesive, and strong adhesive, respectively [37].

### 4.10. MTT Assay

The cytotoxicity of KUNN19-2 on T84 and RAW264.7 cells was determined by the 3-(4,5-dimethyl-thiazol-2-yl)-2,5-diphenyltetrazolium bromide (MTT) assay, as previously described, with a slight modification [38,39]. T84 and RAW264.7 cells were seeded at 4 × 10^4^ cells per well in a 96-well plate for 24 h at 37 °C with 5% CO_2_. Cell culture media were replaced with fresh complete media without FBS and antibiotics for 16–18 h before the assay. Cells were incubated with different MOIs (0 to 20,000) of KUNN19-2 for 24 h at 37 °C with 5% CO_2_. Cell culture media were discarded, and cells were washed twice with sterile PBS. Then, 300 μL of 1 mg/mL MTT solution (Amnesco, Houston, TX, USA) was added to each well and incubated for 3 h at 37 °C with 5% CO_2_. Cell supernatant was discarded, and 150 μL dimethyl sulfoxide (DMSO) (Sigma-Aldrich, Darmstadt, Germany) was added into each well to dissolve the purple-blue formazan precipitate. Plates were wrapped with an aluminum foil and placed on an orbital shaker (Nedtex, Taipei, Taiwan) for 10 min at room temperature. The absorbance at 492 nm was measured by a microplate reader (BioTek Synergy H4 Hybrid Reader, BioTek Instruments Inc., San Diego, CA, USA). The percentage of cell viability was calculated using the following formula: cell viability (%) = [(Abs 492 nm of the treated group-blank)/(Abs 492 nm of the control-blank)] × 100.

### 4.11. Gentamicin Protection Assay for Phagocytic Killing Activity

RAW264.7 cells were seeded in a 24-well plate (approximately 10^6^ cells per well). Cells were pretreated with viable or heat-killed KUNN19-2 at MOI 20 for 24 h at 37 °C with 5% CO_2_. Probiotics were prepared by resuspending bacterial cell pellets in sterile PBS. To promote the flagella expression of *Salmonella*, the important virulence factor of its invasion to mammalian gut epithelium, *Salmonella* overnight culture was sub-cultured in the high-osmolarity LB broth (LB broth supplemented with 300 mM NaCl), as previously described [40]. Then, the pretreated RAW264.7 cells were infected with different *Salmonella* strains at MOI 5. After 1 h of infection, cell culture media were replaced with media supplemented with 100 µg/mL gentamicin sulfate (Sigma-Aldrich, Germany) and incubated at 37 °C with 5% CO_2_ for 90 min to eliminate the extracellular *Salmonella* population. The infected cells were lysed by 1% TritonX-100 (Sigma-Aldrich, Germany), and the recovered intracellular *Salmonella* (colony-forming unit (CFU)/mL) was calculated by a serial ten-fold dilution.

### 4.12. Detection of Macrophage Proinflammatory and Anti-Inflammatory Gene Expressions by Quantitative Polymerase Chain Reaction

Total RNA from the cell culture assay performed in a 6-well plate was extracted using a TRIzol reagent (Invitrogen, Waltham, MA, USA), following the manufacturer’s instruction. The RevertAid First Strand cDNA reagent (Thermo Fisher Scientific, Vilniaus, Lithuania) was used for cDNA synthesis. The list of primer pairs for each targeted gene and *Gapdh* as a housekeeping gene are shown in Supplementary Table S4. The quantitative polymerase chain reaction (qPCR) was performed by the StepOne Plus RT-PCR system using TaqMan^®^ Master mix and ViiA 7 Real-Time PCR system (Applied Biosystems, Carlsbad, CA, USA). Fold change for gene expression was calculated by the comparative threshold cycle (Ct) method, as previously described [23].

### 4.13. Statistical Analysis

The statistical analysis was performed by the GraphPad Prism software V10. Student’s *t*-test was used to analyze the difference between two groups of data, and one-way ANOVA with Turkey’s test was used for the multiple comparisons. * *p* < 0.05 was defined as a statistically significant difference. Bacterial counts and the fold change for mRNA levels were logarithmically transformed before the statistical analysis.

## 5. Conclusions

We reported the in vitro study of anti-*Salmonella* and gut immunity (colonic epithelium and macrophage) modulatory effects of viable (probiotics) and (heat-inactivated) paraprobiotics *L. plantarum* KUNN19-2 against multiple serovars (six isolates) of clinical MDR *Salmonella*. Only probiotic KUNN19-2 can directly inhibit *Salmonella* growth. However, both pro- and paraprobiotic forms of KUNN19-2 activate gut epithelium and macrophage against *Salmonella* serovars in vitro. Paraprobiotic KUNN19-2 should be an alternative treatment against MDR *Salmonella* by host immune modulations. The further in vivo investigation on probiotic and paraprobiotic activities of KUNN19-2 should be performed.

## Data Availability

Please contact the corresponding author for other data requests.

## Supplementary Materials

The following supporting information can be downloaded at https://www.mdpi.com/article/10.3390/ijms26051821/s1. References [41,42,43,44] are cited in the supplementary materials.
