# Supplementary material for: Probiotic, Paraprobiotic, and Postbiotic Activities of Lactiplantibacillus plantarum KUNN19-2 Against Non-Typhoidal Salmonella Serovars
Source: Int J Mol Sci. 2025 Feb 20;26(5):1821. doi: 10.3390/ijms26051821 (PMC11899724; doi:10.3390/ijms26051821)
Supplement: Supplementary file 1 [file ijms-26-01821-s001.zip › ijms-3414297-supplementary.pdf]

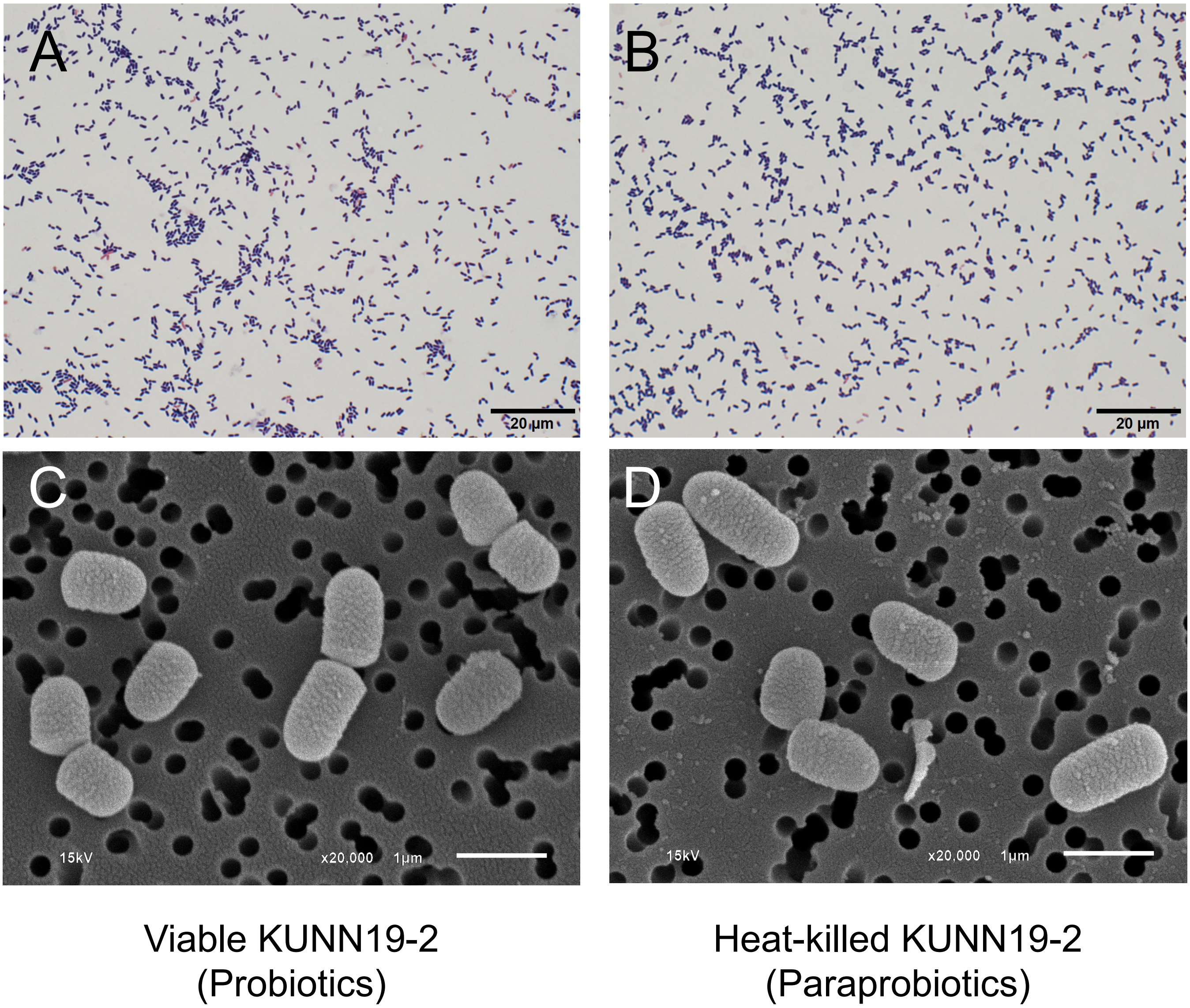
A
B
C
D
20 µm
20 µm
15kV
x20,000
1µm
15kV
x20,000
1µm
Viable KUNN19-2
(Probiotics)
Heat-killed KUNN19-2
(Paraprobiotics)

Figure S1

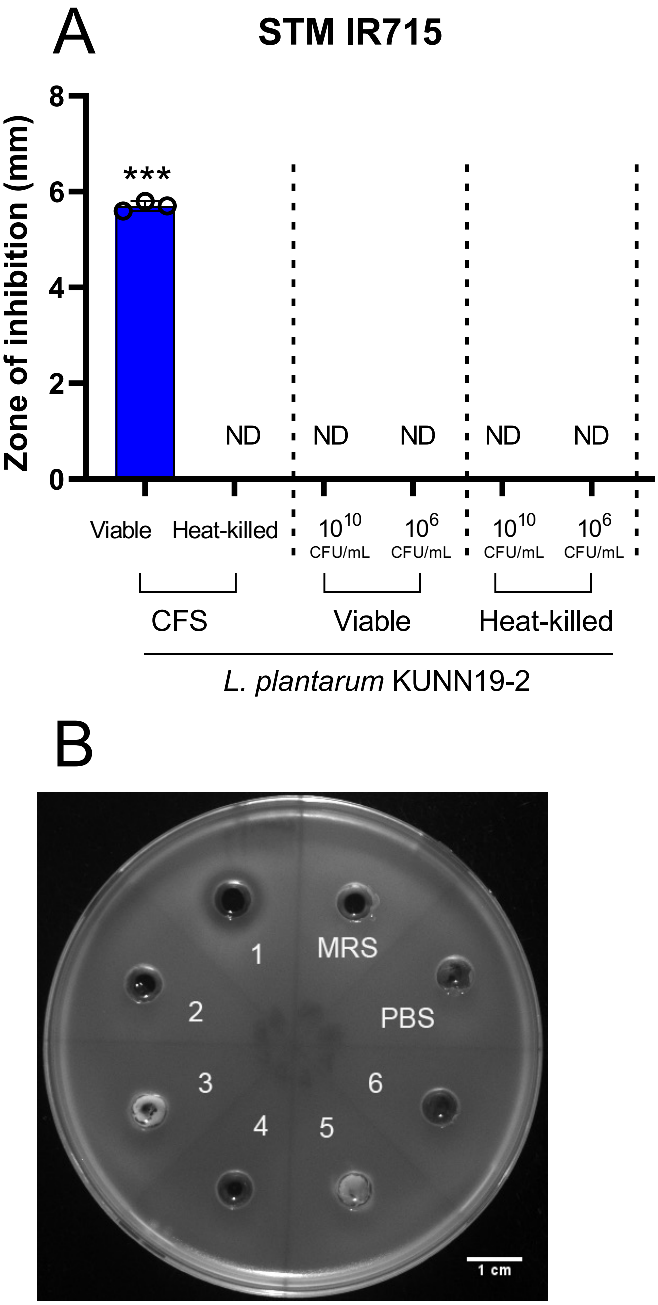
A
STM IR715
Zone of inhibition (mm)
***
ND
ND
ND
ND
ND
Viable
Heat-killed
10^10 CFU/mL
10^6 CFU/mL
10^10 CFU/mL
10^6 CFU/mL
CFS
Viable
Heat-killed
L. plantarum KUNN19-2
B
1
2
3
4
5
6
MRS
PBS
1 cm

Figure S2

Supplementary Table S1. Bacterial strains used in this study

Designation	Species	Serovar	Relevant characteristic	Reference
KUNN19-2	*Lactiplantibacillus plantarum*	N/A	Probiotic isolated from Thai-style fermented pork (Nham). Formerly named *Lactobacillus johnsonii* KUNN19-2 (re-classified in 2024 as *L. plantarum* by 16S rRNA sequencing)	(1)
IR715	*Salmonella enterica*	Typhimurium	Nalidixic acid-resistant derivative of ATCC 14028	(2)
STMC58	*Salmonella enterica*	1,4,[5],12:i-	MDR clinical isolate	This study
STMC81	*Salmonella enterica*	Typhimurium	MDR clinical isolate	This study
STMC101	*Salmonella enterica*	Krefeld	MDR clinical isolate	This study
STMC103	*Salmonella enterica*	Typhimurium	MDR clinical isolate	This study
STMC166	*Salmonella enterica*	Rissen	MDR clinical isolate	This study
STMC177	*Salmonella enterica*	Enteritidis	clinical isolate	This study

Supplementary Table S2. Clinical characteristics of six non-typhoidal salmonellosis (NTS) patients admitted at Maharaj Nakorn Chiang Mai Hospital, Chiang Mai, Thailand, in 2019 and 2022 and the corresponding *Salmonella* isolates

STMC	Year	Source	Serovar	Age (year)	Gender	Sepsis (SIRS)	Hct(%)	Leuk. (cells/mm^3)	Neutro. (cells/mm^3)	Lympho. (cells/mm^3)	*spvB*	*sseI*	*sodCI*	*rpoS*
58	2017	Stool	1,4,[5],12:i-	2	female	Yes	40.2	10700	75.8	14.9	-	+	+	+
81	2017	Stool	Typhimurium	60	male	No	42.4	8600	66.7	27	-	-	+	+
101	2017	Stool	Krefeld	82	female	Yes	29.6	8700	76.3	14.1	-	-	-	+
103	2017	Stool	Typhimurium	72	male	Yes	22	18800	73.1	20.6	-	-	+	+
166	2019	Stool	Rissen	10 months	male	No	27.9	7810	27.6	56.1	-	-	-	+
177	2018	Stool	Enteritidis	19	male	No	N/A	N/A	N/A	N/A	+	+	+	+

M, male; F, female; N/A, Data not available; Hct, hematocrit (normal = 13-18%); Leuk, leukocyte (normal = 5000 – 10000 cells/mm^3); Neutro, neutrophil (normal = 40-74 cells/mm^3) and Lympho, lymphocyte (normal = 19-48 cells/mm^3); +, presence; -, absence

Supplementary Table S3. Antibiotic susceptibility test for six clinical isolates of NTS. Five of six isolates (STMC058, 081, 101, 103, 166) possess the multidrug-resistant (MDR) phenotype. A total of 15 antibacterial agents in 9 categories were used, and the interpretation was done following the CLSI 2020 guideline. R $\geq$ 3 categories are considered MDR. S, susceptible; R, resistant; I, Intermediate

Categories	Agent	*Salmonella enterica* clinical isolates (STMC)					
		058	081	101	103	166	177
Aminoglycosides	Streptomycin (S)	R	R	R	R	R	S
1st and 2nd generation cephalosporins	Cefazolin (KZ)	R	R	R	R	S	S
	Cefuroxime (CXM)	R	R	R	R	S	S
3rd and 4th generation cephalosporins	Cefotaxime (CTX)	R	R	R	R	S	S
	Ceftriaxone (CRO)	R	R	R	R	S	S
	Ceftazidime (CAZ)	I	I	I	R	S	S
	Cefepime (FEP)	R	R	R	R	S	S
Fluoroquinolones	Nalidixic acid (NA)	I	I	I	S	S	R
	Ciprofloxacin (CIP)	S	I	I	I	S	S
Monobactams	Aztreonam (ATM)	R	R	R	R	S	S
Penicillins with β-lactamase inhibitors	Amoxycillin - Clavulanic Acid (AMC)	R	R	R	R	S	S
Tetracyclines	Doxycycline (DO)	R	R	R	I	R	S
	Tetracycline (TE)	R	R	R	R	R	S
Folate pathway antagonist	Trimethoprim-Sulfamethoxazole (SXT)	R	S	S	R	S	S
Penicillins	Ampicillin (AMP)	R	R	R	R	R	S

Supplementary Table S4. The primers used in this study.

Targeted genes	Sequences (5’ → 3’)	References
Mouse *Il10*_Fwd	GGTTGCCAAGCCTTATCGGA	(3)
Mouse *Il10*_Rev	ACCTGCTCCACTGCCTTGCT	
Mouse *Nos2*_Fwd	CCAGCCTTGCATCCTCATTGG	(4)
Mouse *Nos2*_Rev	CCAAACACCAAGCTCATGCGG	
Mouse *Mip2 (Cxcl2)_Fwd*	AGTGAACTGCGCTGTCAATGC	(5)
Mouse *Mip2 (Cxcl2)*_Rev	AGGCAAACTTTTTGACCGCC	
Mouse *Gapdh*_Fwd	TGTAGACCATGTAGTTGAGGTCA	(4)
Mouse *Gapdh* _Rev	AGGTCGGTGTGAACGGATTTG	
*Salmonella spvB*_Fwd	GACTATCTTTCCACAAATGAACCC	(6)
*Salmonella spvB*_Rev	GTATCTATGAGTTGAGTACCTC	
*Salmonella sseI*_Fwd	TCCGCCGATAACCTTATTGTG	(6)
*Salmonella sseI*_Rev	CTGTCATCTGTGATAGTGTCC	
*Salmonella sodCI*_Fwd	TATCGGAGTAATTGTCACCG	(6)
*Salmonella sodCI*_Rev	ACAATATTGTCGCTGGTAGC	
*Salmonella rpoS*_Fwd	TGCTGGCAGAAGACAAACGG	(6)
*Salmonella rpoS*_Rev	TGATTACCTGAGTGCCTACG	

References for Supplementary materials

1. Prommadee P, Garnjanagoonchorn W, de Lange K, Nitisinprasert S. Characterization of Lactobacillus johnsonii KUNN19-2 and Pediococcus pentosaceus KUNNE6-1 isolated from thai-style fermented pork (Nham) for their probiotic properties in the gastrointestinal tract and immunomodulation. Agriculture and Natural Resources. 2012;46(3):440-50.
2. Stojiljkovic I, Baumler AJ, Heffron F. Ethanolamine utilization in Salmonella typhimurium: nucleotide sequence, protein expression, and mutational analysis of the cchA cchB eutE eutJ eutG eutH gene cluster. J Bacteriol. 1995;177(5):1357-66.
3. Xavier MN, Winter MG, Spees AM, Nguyen K, Atluri VL, Silva TM, et al. CD4+ T cell-derived IL-10 promotes Brucella abortus persistence via modulation of macrophage function. PLoS pathogens. 2013;9(6):e1003454.
4. Winter SE, Thiennimitr P, Winter MG, Butler BP, Huseby DL, Crawford RW, et al. Gut inflammation provides a respiratory electron acceptor for Salmonella. Nature. 2010;467(7314):426-9.
5. Winter SE, Thiennimitr P, Nuccio SP, Haneda T, Winter MG, Wilson RP, et al. Contribution of flagellin pattern recognition to intestinal inflammation during Salmonella enterica serotype typhimurium infection. Infection and immunity. 2009;77(5):1904-16.
6. Preziosi MJ, Kandel SM, Guiney DG, Browne SH. Microbiological analysis of nontyphoidal Salmonella strains causing distinct syndromes of bacteremia or enteritis in HIV/AIDS patients in San Diego, California. Journal of clinical microbiology. 2012;50(11):3598-603.
